# Antidepressants and risk of postural hypotension: a self-controlled case series study in UK primary care

**DOI:** 10.3399/BJGP.2024.0429

**Published:** 2025-05-20

**Authors:** Cini Bhanu, Kate Walters, Mine Orlu, Daniel Davis, Reecha Sofat, Irene Petersen

**Affiliations:** Research Department of Primary Care and Population Health, University College London, London; Research Department of Primary Care and Population Health, University College London, London; University College London School of Pharmacy, London; MRC Unit for Lifelong Health and Ageing, University College London, London; Department of Pharmacology and Therapeutics, University of Liverpool, Liverpool; British Heart Foundation, Data Science Centre, Health Data Research UK; Research Department of Primary Care and Population Health, University College London, London

**Keywords:** antidepressants, antidepressive agents, blood pressure, orthostatic hypotension, postural hypotension, primary health care

## Abstract

**Background:**

Antidepressants are associated with postural hypotension (PH), but it is not typically recognised as a common adverse effect. PH is linked with serious complications in older adults, for example, falls, stroke, and cognitive decline. Randomised controlled trials (RCTs) examining antidepressants often exclude older people and do not focus on adverse effects.

**Aim:**

To examine the risk of PH associated with antidepressant use in adults aged ≥60 years in UK primary care.

**Design and setting:**

A self-controlled case series using routinely collected primary care data from the IQVIA Medical Research Database (IMRD) from 1 January 2000 to 31 December 2018.

**Method:**

Data from >41 000 adults aged ≥60 years in IMRD between 1 January 2000 to 31 December 2018 were obtained. Antidepressant prescriptions were determined using code lists based on British National Formulary classification. Risk of PH was examined during four exposure risk periods (90-days pre-prescription; day 1–28; 29–56; and ≥57) compared with periods outside these risk windows.

**Results:**

Among 41 005 people with incident PH in the study period, 8899 (22%) were prescribed a selective serotonin reuptake inhibitor (SSRI); 8313 (20%) were prescribed a tricyclic antidepressant (TCA); and 4656 (11%) were prescribed an ‘other antidepressant’. The authors observed a consistent increased risk of PH in day 1–28 in *all* antidepressant exposure, which reduced thereafter. Risk of PH was highest with SSRIs (incidence rate ratio [IRR] 4.22, 95% confidence interval [CI] = 3.76 to 4.74), followed by ‘other antidepressants’ (IRR 2.17, 95% CI = 1.76 to 2.68), and then TCAs (IRR 2.12, 95% CI = 1.79 to 2.50). Risk of PH reduced from day ≥28 for all antidepressants.

**Conclusion:**

A statistically substantial increased risk of PH was observed with all antidepressants in the first month, particularly SSRIs. Prescribers should be aware of this risk and may consider monitoring PH when initiating antidepressants in older adults.

## Introduction

Postural hypotension (PH) is defined as a reduction in systolic blood pressure (BP) of ≥20 mmHg or diastolic BP of ≥10 mmHg within 3 minutes of assuming an upright posture (or on assuming a head-up position of at least 60 degrees during tilt table testing).^1^ PH is common, affecting around one-third of older people.^2^ It is associated with serious complications in older people.^3^ This includes increased risk of falls;^4^,^5^ myocardial infarction (MI);^6^–^8^ stroke;^3^,^6^ atrial fibrillation;^6^ dementia;^4^,^5^ and mortality^4^,^6^,^7^ related to cerebral and cardiac hypoperfusion. PH has been described as an adverse effect of antidepressants in previous studies with limitations,^9^,^10^ but is rarely recognised in practice. PH is not acknowledged as a side effect of tricyclic antidepressants (TCAs) and is cited as an ‘uncommon’ side effect of selective serotonin reuptake inhibitors (SSRIs) in the British National Formulary (BNF).^11^ Over 87% of older people with depression are prescribed an antidepressant^12^ and this is increasing.^13^ Furthermore, TCAs are widely used for pain and sleep problems.^14^ Most are managed in general practice,^12^ where PH is poorly identified.^15^ There are few guidelines to manage drug-induced PH.^9^,^10^

Most evidence on this topic is from small cross-sectional studies^9^ or randomised controlled trials (RCTs) that either exclude older adults^10^,^12^,^16^ or poorly assess adverse effects of drugs as a secondary outcome to efficacy.^17^

In this study the authors aimed to evaluate the risk of incident PH associated with antidepressant treatment in adults aged ≥60 years in UK primary care using routinely collected data from the IQVIA Medical Research Database (IMRD).

## Method

### Study design and period

This article presents a self-controlled case series (SCCS) study including data from 1 January 2000 to 31 December 2018 (the latest date for which data were available) to examine the associations between antidepressant exposure and adverse PH.

**How this fits in**

Antidepressants are associated with postural hypotension (PH). This is not widely recognised in general practice, where antihypertensives are considered the worst culprits. The present study examined >21 000 older adults and found a striking increased risk of PH with use of all antidepressants (over a four-fold risk with SSRIs) in the first 28 days of initiation.

The SCCS method is used to study the association between a time-varying exposure and an outcome event.^18^ It is an alternative method to cohort and case-control studies and was originally developed to investigate associations between vaccination and acute adverse events.^18^,^19^ Since, this method has been used widely in pharmacoepidemiology^18^ — including many studies examining acute adverse effects of individual drugs.^20^–^22^

The SCCS analysis only uses cases. Therefore, the risk of the outcome is compared during specific periods of drug exposure (exposure risk period) compared to periods outside these risk windows (reference period) to determine whether the risk of the event is greater during drug exposure. Its key strengths are that it only requires cases, and individuals act as their own control. SCCS compares risk *within* individuals rather than between individuals. This eliminates fixed confounding, for example, sex, ethnicity, social deprivation, and genetic factors.^23^,^24^ It offers a method of assessing adverse drug risks in complex older adults with comorbidity and polypharmacy more representative of real-world prescribing, who would have otherwise been excluded from RCTs.

### Data source

This study used general practice data from anonymised electronic healthcare records contributing to IMRD, which includes >18 million patients from >750 general practices.^25^ These broadly represent UK practices in terms of age, sex, practice size, geographical distribution, and sociodemographic characteristics.^26^

During the study period, most information was systematically recorded using the Read classification coding system.^27^ In the UK, GPs often use the BNF to guide prescribing.^11^ Prescription data are coded automatically when entered and are essentially complete (though this does not include drugs prescribed in hospital). Prescription data can be linked to diagnoses and other clinical information^28^ in IMRD.

### Inclusion criteria

For participants included in the source population, the observation period for each individual started at the latest of:

- 1 January 2000;
- their registration date;
- date of recording of data at acceptable quality and mortality reporting levels; or
- their sixtieth birthday; plus
- 12 months of continuous registration in IMRD.

Eligible participants were those with both the exposure of interest (antidepressant prescription) *and* the outcome of interest (incident PH) during the study period (1 January 2000 to 31 December 2018).

The end of the observation period for each individual was the earliest of:

- 31 December 2018;
- last data collection date for the practice;
- transfer out of the practice;
- death; or
- their hundredth birthday.

### Exposure

Antidepressant prescriptions were determined using drug code lists developed using established methods^29^ based on the following BNF sub-chapters:

- Chapter 4.3.1: Tricyclic and related antidepressant drugs (for example, amitriptyline).
- Chapter 4.3.3: Selective serotonin re-uptake inhibitors (for example, sertraline).
- Chapter 4.3.4: Other antidepressant drugs (for example, mirtazapine).

Prescription data in IMRD has been grouped according to these sub-chapters. The ‘Other antidepressant drugs’ sub-chapter included: agomelatine, duloxetine, flupentixol, mirtazapine, nefazodone, oxitriptan, reboxetine, tryptophan, venlafaxine, and vortioxetine.

A prescription was considered an eligible prescription if it was the first prescribed antidepressant for the patient and it occurred during the study period. A prescription episode was deemed to be continuous if there was a ≤90-day gap between consecutive prescriptions. This method has been used in previous studies examining antidepressants.^30^ The start and end dates of drug exposure were determined for each individual based on their first continuous episode of prescriptions only. The end of drug exposure was defined as the last prescription date of the continuous episode plus 30 days, to account for the final prescription duration.^24^

### Drug exposure risk periods

#### Pre-exposure risk period

**Figure 1:**
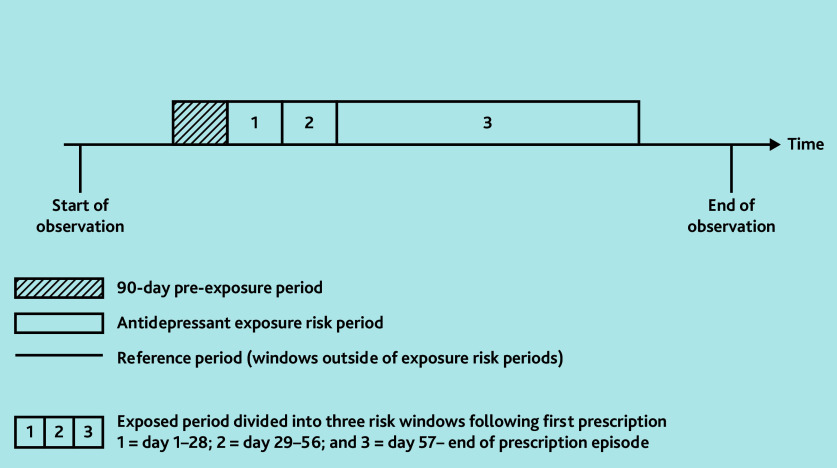
Illustration of self-controlled case series study design. The figure illustrates a single individual with antidepressant exposure during their observation period, demonstrating the different exposure risk periods.

A key SCCS assumption is that the occurrence of an event (PH) should not appreciably affect subsequent exposures (antidepressant prescriptions). In this case, a recorded diagnosis of PH may temporarily reduce the likelihood of a clinician prescribing an antidepressant afterwards and may potentially produce biased estimates.^19^ Therefore, the authors included a 90-day pre-exposure period before first prescription (Figure 1) to account for this assumption.

#### Initiation risk period (day 1–28; day 29–56)

The initiation period of an antidepressant was defined as day 1–28 from first prescription (Figure 1). This timeframe was considered most likely to reflect the development of drug-induced PH, as opposed to the development of PH related to other causes. The researchers included an additional initiation period between day 29–56, which accounted for a larger window for patients to present to the GP with side effects or for the new medication to be reviewed.

#### Continuation risk period (day ≥57)

The continuation period of antidepressant exposure was defined as time from day 57 onwards to the end of the prescription episode (this was different for each individual) (Figure 1). The end of the prescription episode was defined as the last prescription date plus 30 days (to account for the final prescription exposure).

#### Reference period

The reference periods were periods outside the risk windows described above, without antidepressant exposure (Figure 1).

### Outcome

The outcome was defined as the participant’s first recorded PH diagnosis that occurred during the observation period. PH was identified through a specific code list that included four Read codes with high certainty of a validated diagnosis:

- ‘O/E — BP reading: postural drop’ (medcode 2468.00);
- ‘orthostatic hypotension’ (G870.00);
- ‘postural hypotension’ (G870.11); and
- ‘Parkinsonism with orthostatic hypotension’ (F130300).^15^

### Statistical analysis

Conditional Poisson regression was used to calculate incident rate ratios (IRRs) comparing the risk of PH during periods of exposure risk with periods outside of these risk windows. Adjustments for age in 5-year age bands: 60–64; 65–69; 70–74; 75–79; 80–84; 85–89; 90–94; and 95–99 years were made.

### Patient and public involvement (PPI)

A group of older representatives and clinical pharmacists were involved in exploring the potential implications of the results and limitations, which are presented in the Discussion section of this article.

## Results

In IMRD, there were 2 597 077 people aged ≥60 years who were eligible as the source population between 1 January 2000 and 31 December 2018. Among these, 41 005 (2%) people had a first recorded PH diagnosis within the observation period; and 19 979 (49%) were male and 21 026 (51%) were female.

Of the eligible patients with PH, 8313 (20%) had a first recorded TCA prescription; 8899 (22%) had a first recorded SSRI prescription; and 4656 (11%) had a first recorded prescription of an ‘other antidepressant’ in the study period (Table 1).

**Table 1:** Patient demographics of eligible cohort with incident postural hypotension and incident antidepressant prescription between 1 January 2000 and 31 December 2018

Antidepressant exposure	TCA, *N* = 41 005	SSRI, *N* = 41 005	Other antidepressant, *N* = 41 005
**Overall, ** ***n*** ** (%)**	8313 (20)	8899 (22)	4656 (11)

**Sex, ** ***n*** ** (%)**			
Male	3780 (45)	3848 (43)	1970 (42)
Female	4533 (55)	5051 (57)	2686 (58)

**Age band on entry, years, ** ***n*** ** (%)**			
60–64	2414 (29)	2157 (24)	1404 (30)
65–69	1740 (21)	1691 (19)	919 (20)
70–74	1761 (21)	1945 (22)	990 (21)
75–79	1354 (16)	1667 (19)	775 (17)
80–84	764 (9)	1004 (11)	392 (8)
85–89	222 (3)	353 (4)	144 (3)
90–94	52 (1)	78 (1)	29 (1)
95–99	6 (0.1)	4 (0.04)	3 (0.1)

SSRI = selective serotonin reuptake inhibitor. TCA = tricyclic antidepressant.

### Selective serotonin reuptake inhibitors

**Figure 2:**
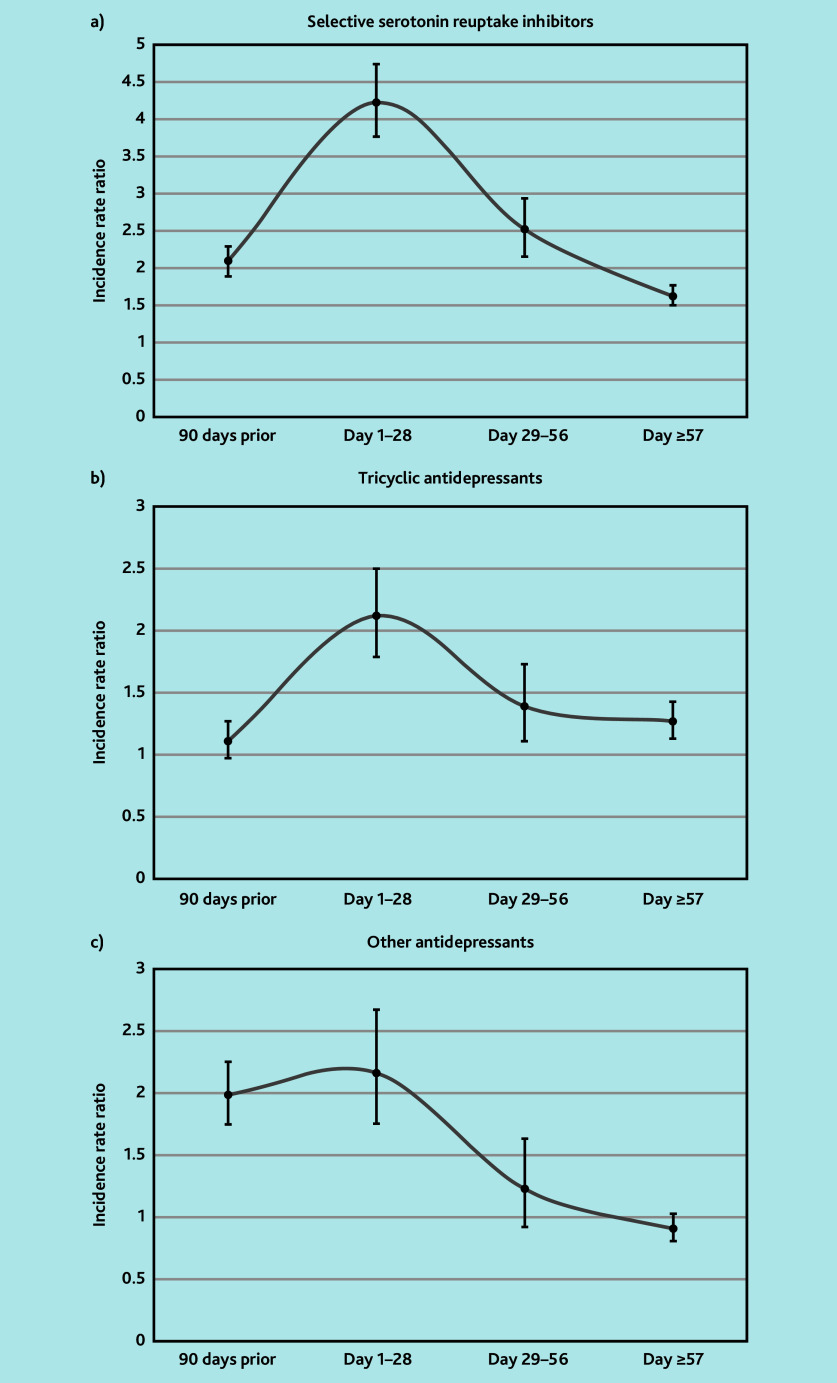
Graphical representation of incidence rate ratios and 95% confidence intervals in the risk periods for tricyclic antidepressants, selective serotonin reuptake inhibitors, and other antidepressants to illustrate similar trends in timing of incident postural hypotension (peak in day 1–28 risk period).

Among SSRIs, the main prescriptions were citalopram (35%), sertraline (17%), and fluoxetine (20%) (data not shown). The authors observed increased risk of PH with SSRI exposure during days 1–28 (IRR 4.22, 95% confidence interval [CI] = 3.76 to 4.74), this risk remained increased but to a lesser extent during days 29–56 (IRR 2.52, 95% CI = 2.15 to 2.94) and during days 57–end of episode (IRR 1.62, 95% CI = 1.50 to 1.77) (Table 2 and Figure 2). An increased risk was also found during the 90-day pre-exposure period (IRR 2.10, 95% CI = 1.89 to 2.29).

**Table 2:** Incidence rate ratios for postural hypotension associated with TCAs, SSRIs, and ‘other antidepressants’

Reference period	Events, *n*	Total person–years	IRR (95% CI)
**Tricyclic antidepressants, ** ***N*** ** = 8313**	7356	90 318	Ref
90-day pre-exposure period	223	2014	1.11 (0.97 to 1.27)
Day 1–28	141	658	2.12 (1.79 to 2.50)
Day 29–56	81	582	1.39 (1.11 to 1.73)
Day 57–end of prescribing episode	6303	72 132	1.27 (1.13 to 1.43)

**SSRIs, ** ***N*** ** = 8899**	6861	87 880	Ref
90-day pre-exposure period	453	2159	2.10 (1.89 to 2.29)
Day 1–28	302	701	4.22 (3.76 to 4.74)
Day 29–56	161	632	2.52 (2.15 to 2.94)
Day 57–end of prescribing episode	1156	8367	1.62 (1.50 to 1.77)

**Other antidepressant, ** ***N*** ** = 4656**	3782	46 941	Ref
90-day pre-exposure period	258	1137	1.99 (1.75 to 2.26)
Day 1–28	92	365	2.17 (1.76 to 2.68)
Day 29–56	46	326	1.23 (0.92 to 1.64)
Day 57–end of prescribing episode	478	4687	0.91 (0.81 to 1.03)

IRR = incidence rate ratio. SSRI = selective serotonin reuptake inhibitor. TCA = tricyclic antidepressant.

### Tricyclic antidepressants

Among TCAs, amitriptyline accounted for most prescriptions (81%) (data not shown). The authors observed an increased risk of PH with TCA exposure during days 1–28 (IRR 2.12, 95% CI = 1.79 to 2.50). This risk remained increased but to a lesser extent during days 29–56 (IRR 1.39, 95% CI = 1.11 to 1.73) and during days 57–end of episode (IRR 1.27, 95% CI = 1.13 to 1.43). No increased risk was found during the 90-day pre-exposure period (IRR 1.11, 95% CI = 0.97 to 1.27) (Table 2 and Figure 2).

### Other antidepressant drugs

In this group, the majority of prescriptions were mirtazapine (64%), duloxetine (12%), and venlafaxine (12%) (data not shown). An increased risk of PH associated with other antidepressant exposure during days 1–28 (IRR 2.17, 95% CI = 1.76 to 2.68) was observed. There was no significant increased risk of PH after this period. An increased risk was also found during the 90-day pre-exposure window (IRR 1.99, 95% CI = 1.75 to 2.26) (Table 2 and Figure 2).

Figure 2 illustrates similar trends in the timing of incident PH related to the risk periods in all antidepressant groups, where the increased risk of PH peaks in the day 1–28 period, then falls either side.

## Discussion

### Summary

The presented SCCS analysis of antidepressants and PH, covering >21 000 older adults (a real-world population that might have otherwise been excluded from RCTs), showed a statistically significant increased risk of PH within the first 28 days of all antidepressant exposure. The groups examined included: TCAs, SSRIs, and ‘other antidepressants’ (mirtazapine, duloxetine, and venlafaxine). Overall, risk of PH was highest with SSRIs (IRR 4.22, 95% CI = 3.76 to 4.74), followed by ‘other antidepressants’ (IRR 2.17 (95% CI = 1.76 to 2.68) and TCAs (IRR 2.12, 95% CI = 1.79 to 2.50). The risk of PH reduced from day 28 in all groups. Additionally, the authors found a statistically significant increased risk of PH in the 90-day pre-exposure period for SSRIs and ‘other antidepressants’, which was not observed with TCAs.

### Strengths and limitations

To the authors’ knowledge, this is the first study to explore antidepressants and PH using a large primary care database including a complex older population. There are several notable strengths. IMRD offered an adequate sample size of >41 000 PH cases and almost complete prescription data. Furthermore, RCTs of drugs are powered to evaluate efficacy of the drugs — not adverse effects, which requires much larger sample sizes. Thus, most trials are known to poorly assess adverse effects,^17^ of which PH is often not considered or measured consistently.^31^ RCTs also often exclude older people with multimorbidity and polypharmacy, and those from underserved groups, therefore adverse effects and potential harms may be attenuated.^32^ The SCCS analysis offered an invaluable way to closely examine the risk of PH with antidepressant use during specific periods of exposure, in an older population, and accounting for all time-invariant confounding.

There are some limitations. For example, this study did not have a sufficiently large sample size to examine effects by individual drug or dose and thus the authors evaluated the effects by higher-level drug categories (BNF sub-chapters).

Additionally, some prescriptions issued in primary care may never be dispensed and used. There may have been individuals in the present study who were prescribed a drug but did not take the medication. Thus, the presented estimates might slightly underestimate the effect. On the other hand, there may have been a small number of prescriptions prescribed in secondary care settings, for example, psychiatry clinics, which will not have been captured in primary care records.

While the SCCS method accounts for fixed confounding, the researchers could not control for the development of new conditions, changes in severity of the underlying condition, or exposure to new medications, independently associated with PH. However, this has been mitigated somewhat through the use of short well-defined risk periods. There was also potential for confounding by indication, that is, whether the indication for the drug (underlying illness) has increased the risk of PH, rather than the drug itself. For SSRIs and ‘other antidepressants’, most patients are likely to have depression, while TCAs are often prescribed for other reasons such as neuropathic pain and insomnia.^14^ The authors observed an interesting pattern of an increased ‘background risk’ during the 90-day pre-exposure period for SSRIs and ‘other antidepressants’ that was not seen in TCAs. This could demonstrate a background risk related to underlying depression (disentangled from the acute risk of drug exposure) since depression is independently associated with PH.^33^ Whereas the common indications for TCAs in the UK is not depression, but problems such as neuropathic pain, insomnia, and migraine^14^ (which are not independently associated with PH).

The SCCS analysis is reliant on the timely recording of acute events.^18^ However, in this primary care dataset there may have been some time lag between a patient experiencing PH and it being recorded in the GP record. On the other hand, there may also be some potential bias related to patients being more likely to report and attribute symptoms of PH to a drug when it is initiated, rather than if symptoms emerge later, which was flagged by our PPI group. However, it is noticeable that the trends observed are consistent across all three antidepressant groups. The researchers also used narrow windows of exposure increasing confidence that the effects observed in this study are related to antidepressant initiation.

### Comparison with existing literature

The authors’ finding that SSRIs were associated with the highest risk of PH in this study — a four-fold increased risk of PH during the days 1–28 exposure risk period — was surprising. SSRIs are considered first-line for depression in older adults,^13^ generally considered a ‘safer’ antidepressant,^33^ and are the most frequently prescribed drug in older people.^13^,^31^ A recent systematic review on drug-induced PH in RCTs found that SSRIs were not associated with PH.^10^ However, this evidence was based on trials in a younger population (with an average age of 57 years versus a mean age of 71 years in this study).

More aligned with the findings of the study presented in this article, Briggs *et al* found a doubled risk of PH with SSRIs (odds ratio 2.11, 95% CI = 1.25 to 3.57) in a cross-sectional study.^34^ SSRIs have mechanisms of action that can affect postural BP. This includes inhibition of calcium channels and vasoconstriction, and a reduction in heart rate. These mechanisms could also be potentiated in an older cohort.^35^

It has been reported that SSRIs cause PH less frequently than TCAs.^9^,^10^ However, this may be when TCAs are used at higher doses for depression (which is now less common in clinical practice) rather than at lower doses for insomnia and pain, which are reflective of current use.^14^

A clear trend with all antidepressant groups in the present study was that the risk of PH was highest during the first 1–28 days of initiation. This is reminiscent of the acute ‘first-dose phenomenon’ that was first documented for prazosin (an alpha-adrenoreceptor blocker) by Graham *et al* in 1976,^36^ characterised by severe symptomatic PH that usually occurred within 90 minutes after a first dose. A recent SCCS study on tamsulosin examining adverse hypotension also found the first 8 weeks following initiation was a high-risk period, similar to the results presented in this article.^37^

PH induced by antidepressants is related to the action of several neurotransmitter systems on BP regulation.^33^ Antidepressant side effects generally occur within an acute 2–4-week window following initiation.^38^ The manifestation of drug-induced PH may also emerge more rapidly in older people owing to differences in pharmacokinetics.^39^ Metabolic changes related to ageing, for example, changes in liver mass and renal function, could affect metabolism of the drug resulting in higher circulating concentrations.^39^,^40^ Pharmacodynamic sensitivity may also increase in older people.^39^

### Implications for research and practice

PH is not listed as an adverse effect in the BNF or Summary of Product Characteristics for TCAs or ‘other antidepressants’, and is cited as an uncommon side effect for SSRIs.^11^ The authors recommend PH should be listed under cautions for older people and as a side effect for TCAs in drug formularies.

Late-life depression is common, and often the decision to prescribe an antidepressant has carefully considered the balance between risk and benefit in the older patient.^12^,^13^ Findings from the presented study support safe prescribing of antidepressants where indicated. However, the authors recommend that prescribers are aware of the increased risk of PH when initiating antidepressants in older adults, particularly SSRIs. Prescribers may consider monitoring for PH in high-risk patients, for example, those at risk of falls or at risk of PH for other reasons, such as comorbid diabetes or Parkinson’s disease. Future clinical trials on antidepressants should consider assessing PH as part of adverse effect profiles. Further studies are needed to determine whether there are differences in drug-induced PH within drug class, by dose and underlying comorbidity.

## Data Availability

Dataset is not publicly available.

## References

[1] Freeman R, Wieling W, Axelrod FB (2011). Consensus statement on the definition of orthostatic hypotension, neurally mediated syncope and the postural tachycardia syndrome. Clin Auton Res.

[2] McDonagh STJ, Mejzner N, Clark CE (2021). Prevalence of postural hypotension in primary, community and institutional care: a systematic review and meta-analysis. BMC Fam Pract.

[3] Saedon NI, Pin Tan M, Frith J (2020). The prevalence of orthostatic hypotension: a systematic review and meta-analysis. J Gerontol A Biol Sci Med Sci.

[4] Clark CE, Thomas D, Warren FC (2018). Detecting Risk Of Postural hypotension (DROP): derivation and validation of a prediction score for primary care. BMJ Open.

[5] Duggan E, Romero-Ortuno R, Kenny RA (2019). Admissions for orthostatic hypotension: an analysis of NHS England Hospital Episode Statistics data. BMJ Open.

[6] Ricci F, De Caterina R, Fedorowski A (2015). Orthostatic hypotension: epidemiology, prognosis, and treatment. J Am Coll Cardiol.

[7] Fedorowski A, Stavenow L, Hedblad B (2010). Orthostatic hypotension predicts all-cause mortality and coronary events in middle-aged individuals (The Malmo Preventive Project). Eur Heart J.

[8] Gupta V, Lipsitz LA (2007). Orthostatic hypotension in the elderly: diagnosis and treatment. Am J Med.

[9] Rivasi G, Rafanelli M, Mossello E (2020). Drug-related orthostatic hypotension: beyond anti-hypertensive medications. Drugs Aging.

[10] Bhanu C, Nimmons D, Petersen I (2021). Drug-induced orthostatic hypotension: A systematic review and meta-analysis of randomised controlled trials. PLoS Med.

[11] National Institute for Health and Care Excellence (2025). British National Formulary (BNF).

[12] Frost R, Beattie A, Bhanu C (2019). Management of depression and referral of older people to psychological therapies: a systematic review of qualitative studies. Br J Gen Pract.

[13] Giovannini S, Onder G, van der Roest HG (2020). Use of antidepressant medications among older adults in European long-term care facilities: a cross-sectional analysis from the SHELTER study. BMC Geriatr.

[14] Coupland C, Dhiman P, Morriss R (2011). Antidepressant use and risk of adverse outcomes in older people: population based cohort study. BMJ.

[15] Bhanu C, Petersen I, Orlu M (2023). Incidence of postural hypotension recorded in UK general practice: an electronic health records study. Br J Gen Pract.

[16] Florisson S, Aagesen EK, Bertelsen AS (2021). Are older adults insufficiently included in clinical trials?-An umbrella review. Basic Clin Pharmacol Toxicol.

[17] Junqueira DR, Phillips R, Zorzela L (2021). Time to improve the reporting of harms in randomized controlled trials. J Clin Epidemiol.

[18] Whitaker HJ, Farrington CP, Spiessens B, Musonda P (2006). Tutorial in biostatistics: the self-controlled case series method. Stat Med.

[19] Petersen I, Douglas I, Whitaker H (2016). Self controlled case series methods: an alternative to standard epidemiological study designs. BMJ.

[20] Chui CSL, Cheung KS, Brown JP (2023). Proton pump inhibitors and myocardial infarction: an application of active comparators in a self-controlled case series. Int J Epidemiol.

[21] Douglas IJ, Langham J, Bhaskaran K (2013). Orlistat and the risk of acute liver injury: self controlled case series study in UK Clinical Practice Research Datalink. BMJ.

[22] Mansfield KE, Douglas IJ, Nitsch D (2018). Acute kidney injury and infections in patients taking antihypertensive drugs: a self-controlled case series analysis. Clin Epidemiol.

[23] Dave S, Petersen I (2009). Creating medical and drug code lists to identify cases in primary care databases. Pharmacoepidemiol Drug Saf.

[24] Ikuta K, Nakagawa S, Yamawaki C (2022). Use of proton pump inhibitors and macrolide antibiotics and risk of acute kidney injury: a self-controlled case series study. BMC Nephrol.

[25] IQVIA (2020). IQVIA medical research data: improving patient outcomes with evidence-based research.

[26] Blak BT, Thompson M, Dattani H, Bourke A (2011). Generalisability of The Health Improvement Network (THIN) database: demographics, chronic disease prevalence and mortality rates. Inform Prim Care.

[27] Chisholm J (1990). The Read clinical classification. BMJ.

[28] Zhang F, Mamtani R, Scott FI (2016). Increasing use of prescription drugs in the United Kingdom. Pharmacoepidemiol Drug Saf.

[29] Hardoon S, Hayes JF, Blackburn R (2013). Recording of severe mental illness in United Kingdom primary care, 2000–2010. PLoS One.

[30] Jeffery A, Bhanu C, Walters K (2023). Polypharmacy and antidepressant acceptability in comorbid depression and type 2 diabetes: a cohort study using UK primary care data. Br J Gen Pract.

[31] Bhanu C, Petersen I, Orlu M (2024). Drug-induced orthostatic hypotension: cluster analysis of co-prescription patterns in older people in UK primary care. Pharmacoepidemiol Drug Saf.

[32] Ruiter R, Burggraaf J, Rissmann R (2019). Under-representation of elderly in clinical trials: an analysis of the initial approval documents in the Food and Drug Administration database. Br J Clin Pharmacol.

[33] Calvi A, Fischetti I, Verzicco I (2021). Antidepressant drugs effects on blood pressure. Front Cardiovasc Med.

[34] Briggs R, Carey D, McNicholas T (2018). The association between antidepressant use and orthostatic hypotension in older people: a matched cohort study. J Am Soc Hypertens.

[35] Lavan AH, Gallagher P (2016). Predicting risk of adverse drug reactions in older adults. Ther Adv Drug Saf.

[36] Graham RM, Thornell IR, Gain JM (1976). Prazosin: the first-dose phenomenon. Br Med J.

[37] Bird ST, Delaney JAC, Brophy JM (2013). Tamsulosin treatment for benign prostatic hyperplasia and risk of severe hypotension in men aged 40–85 years in the United States: risk window analyses using between and within patient methodology. BMJ.

[38] Kelly K, Posternak M, Alpert JE (2008). Toward achieving optimal response: understanding and managing antidepressant side effects. Dialogues Clin Neurosci.

[39] Gutsmiedl K, Krause M, Bighelli I (2020). How well do elderly patients with major depressive disorder respond to antidepressants: a systematic review and single-group meta-analysis. BMC Psychiatry.

[40] Berger SI, Iyengar R (2011). Role of systems pharmacology in understanding drug adverse events. Wiley Interdiscip Rev Syst Biol Med.

